# HYPOTHESIS: Do LRIG Proteins Regulate Stem Cell Quiescence by Promoting BMP Signaling?

**DOI:** 10.1007/s12015-022-10442-9

**Published:** 2022-08-15

**Authors:** Carl Herdenberg, Håkan Hedman

**Affiliations:** Department of Radiation Sciences, Oncology, Umeå University, Umeå, Sweden

**Keywords:** Stem cell, Quiescence, LRIG1, BMP, EGF

## Abstract

Leucine-rich repeats and immunoglobulin-like domains (LRIG) proteins are evolutionarily conserved integral membrane proteins. Mammalian LRIG1 regulates stem cell quiescence in various tissue compartments, including compartments in the epidermis, oral mucosa, intestines, neural system, and incisors. The planarian LRIG1 homolog regulates the quiescence of multipotent neoblasts. The mechanism through which LRIG proteins regulate stem cell quiescence has not been well documented, although it is generally assumed that LRIG1 regulates the epidermal growth factor receptor (EGFR) or other receptor tyrosine kinases. However, *Lrig*-null (*Lrig1-/-;Lrig2-/-;* and *Lrig3-/-*) mouse embryonic fibroblasts (MEFs) have been recently found to exhibit apparently normal receptor tyrosine kinase functions. Moreover, bone morphogenetic protein (BMP) signaling has been shown to depend on LRIG1 and LRIG3 expression. BMPs are well-known regulators of stem cell quiescence. Here, we hypothesize that LRIG1 might regulate stem cell quiescence by promoting BMP signaling.

**Graphical abstract:**

**HYPOTHESIS:** Based on recent findings, it is hypothesized that LRIG1 regulates stem cell quiescence in mammalian tissues as well as in planarian neoblasts by promoting BMP signaling.

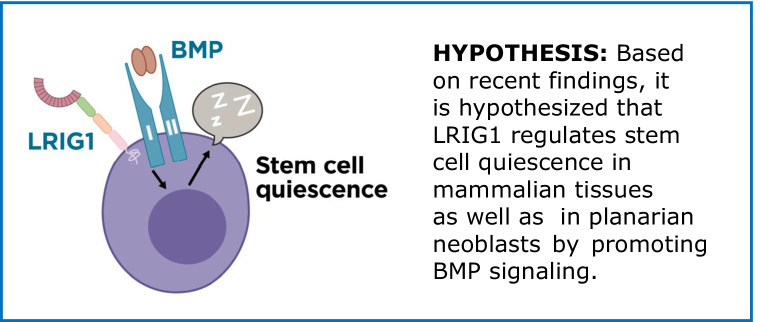

**Supplementary Information:**

The online version contains supplementary material available at 10.1007/s12015-022-10442-9.

## Background

Recent reports have shown that transmembrane protein leucine-rich repeats and immunoglobulin-like domains (LRIG) 1 is both a stem cell marker and a regulator of stem cell quiescence. LRIG1 belongs to the LRIG family of transmembrane proteins, which also includes LRIG2 and LRIG3 [1–4]. Most studies on the role played by LRIG1 in stem cell biology have suggested or assumed that LRIG1 function is mediated through the negative regulation of the epidermal growth factor (EGF) receptor (EGFR) ([for reviews see, [5–8]), although the validity of this assumption has been questioned [9]. Notably, genetically engineered *Lrig*-null (i.e., *Lrig1*^*−/−*^*;Lrig2*^*−/−*^*;* and *Lrig3*^*−/−*^) mouse embryonic fibroblasts (MEFs) were recently found to exhibit apparently normal receptor tyrosine kinase signaling activity [10], challenging the prevailing idea that the primary function of LRIG1 is regulation of receptor tyrosine kinases. Moreover, mammalian LRIG1 and LRIG3 have been shown to promote bone morphogenetic protein (BMP) signaling [10], a pleiotropic signaling pathway known for the important roles it plays in various biological processes, including the maintenance of stem cell quiescence. In the present commentary, we put forth the hypothesis that LRIG proteins might regulate stem cell quiescence primarily through the promotion of BMP signaling, not other mechanisms. This new LRIG paradigm may be relevant to the study of stem cell systems and to the understanding of other physiological processes and diseases in which LRIG proteins play a role.

## Stem Cell Quiescence

Adult tissue homeostasis is maintained by the balanced proliferation and differentiation of tissue-specific stem cells. Tissue stem cells are defined by their ability to self-renew and give rise to the different cell lineages in respective tissues [11]. Therefore, stem cell numbers need to be tightly controlled, such as through the precise regulation of cell proliferation rates. In fact, most adult stem cells are not actively proliferating; in contrast, they remain in a ‘quiescent’ state, which is characterized by reversible exit from the cell cycle, a small cell size, altered metabolism, low RNA content, and reduced protein synthesis [12–14]. Although quiescence is not a defining feature of stem cells [15, 16], it has been argued that the quiescent state prevents proliferative ‘exhaustion’ of stem cell pools and protects stem cell lineages from accumulating mutations and other lesions that might impair their long-term function [15]. Quiescent stem cells have been extensively documented in the hematopoietic system, skeletal muscle, nervous system, skin, and hair follicles, whereas in rapidly regenerating intestines, the presence of quiescent stem cells is less clear (e.g., [17, 18]). The molecular cues and signaling pathways that regulate stem cell quiescence remain incompletely understood.

## LRIG1 and Stem Cell Quiescence

The first indication that LRIG1 is a stem cell marker that regulates stem cell quiescence was presented in 2006 by Jensen and Watt, who showed that certain quiescent epidermal stem cells express and are regulated by LRIG1 [19]. Subsequently, LRIG1 was shown to regulate stem cell quiescence in several additional tissues. Here, we discuss five mammalian tissues and one invertebrate organism in which LRIG1 protein expression has been shown or has been suggested to be both a marker of stem cells and regulator of their quiescence.

In **the epidermis**, Lrig1 expression marks quiescent epidermal interfollicular stem cells that are maintained in a quiescent state [19, 20]. Hence, the genetic ablation of *Lrig1* in mice resulted in epidermal hyperplasia [21] as a consequence of the increased proliferation of interfollicular epidermal stem cells located in the junctional zone of hair follicles [20]. In both humans and mice, the epidermis includes several different interconvertible stem cell compartments, and in addition to interfollicular stem cells, these compartments comprise hair follicle stem cells and sebaceous gland stem cells. In sebaceous glands, Lrig1 marks a stem cell population that is highly proliferative and not quiescent [22], which shows that Lrig1 is not universally associated with cell quiescence. However, in the current commentary, we focused on examples in which Lrig1 has been shown to regulate stem cell quiescence, including interfollicular stem cells in the adult epidermis.

In **the hard palate of the oral mucosa**, Lrig1 marks a population of infrequently dividing, i.e., quiescent, stem cells [23]. Interestingly, when the palate is wounded and during the subsequent induction of epithelial cell proliferation, Lrig1 expression is downregulated. In contrast, the genetic inactivation of *Lrig1* results in the hyperproliferation of these stem cells. Hence, Lrig1 is both a marker of oral hard palate epithelial stem cells and a regulator of their quiescence.

LRIG1 also plays an important role in **intestinal homeostasis**. For example, depending on the mouse genetic background, *Lrig1*-knockout animals have shown either excessive postnatal proliferation of intestinal cells that led to the death of the mouse around postnatal day 10 [24] or a more subtle phenotype, with an increased incidence of duodenal tumors observed in mice from 3 to 12 months old [25, 26]. A strong phenotypic dependency on the genetic background is common for mutations in signaling-related genes, as was first shown by Sibilia and Wagner for *Egfr* [27]. In the mutant mouse model showing lethality, *Lrig1* ablation-induced intestinal hyperplasia resulted from an expansion of the intestinal stem cell compartment. The identity and characteristics of the intestinal Lrig1-expressing cell populations remain controversial. For example, although Powell and coworkers showed that Lrig1 marks a relatively rare and largely quiescent crypt stem cell population that is distinct from the highly proliferative Lgr5 + stem cell population [25], Wong and coworkers showed that Lrig1 is expressed by as many as one-third of all crypt cells, including the highly proliferative Lgr5 + stem cells [24]. The reasons for the discrepancy between these two studies are not fully understood but may include the use of different mouse strains or different reagents to assess Lrig1 positivity [28] as well as different definitions (thresholds) of quiescence and Lrig1 positivity. Nevertheless, at least some intestinal stem cells expressed Lrig1, and Lrig1 suppressed the proliferation of these, regardless of whether they were quiescent or not.

In **the mouse brain,** Lrig1 controls the transition of rapidly dividing embryonic neural precursor cells to quiescent postnatal neural stem cells [29]. In the adult mouse brain, Lrig1 marks a population of quiescent neural stem cells located in the wall of the lateral ventricle [30]. When *Lrig1* is genetically inactivated, neural stem cells show enhanced proliferation and impaired exit from the cell cycle [29, 31]. Ectopic overexpression of Lrig1, on the other hand, slows the proliferation of neural stem cells *in vitro* [29, 31]. Hence, Lrig1 is both a marker of quiescent adult neural stem cells and an important regulator of their quiescence.

**Incisors** are continuously renewed during the lifetime of a mouse. A gene coexpression analysis with label retention and lineage tracing experiments revealed that *Lrig1* marks relatively quiescent and long-lived stem cells in mouse incisors as well as the inner periodontal mesenchyme [32]. These *Lrig1*-positive stem cell populations are relatively quiescent; however, the functional role played by *Lrig1* in maintaining the quiescent state of mouse incisor stem cells has not yet been investigated.

**Planarian flat worms** show a remarkable regenerative capacity. In fact, after an injury, all adult planarian tissues can be regenerated through the activation of a population of adult stem cells called neoblasts [33]. Recently, a subpopulation of quiescent neoblasts, characterized by small size, slow cell cycling, and low levels of RNA, was discovered in the planarian *Schmidtea mediterranea* [34]. These slow-cycling neoblasts express high levels of the planarian *LRIG1* ortholog, *Lrig1*, which is needed for maintaining quiescence. Therefore, knocking down *Lrig1* expression results in hyperproliferation and subsequent depletion of this neoblast stem cell population [34]. Hence, *Lrig1* is required to inhibit the proliferation of quiescent neoblasts in *S. mediterranea* during homeostasis. This intriguing discovery suggests that an ancient LRIG1 homolog regulated stem cell quiescence more than 800 million years ago, the time in which last common planarian and mammalian ancestor lived [35].

## LRIG1 is a Conserved Regulator of BMP Signaling

**Fig. 1:**
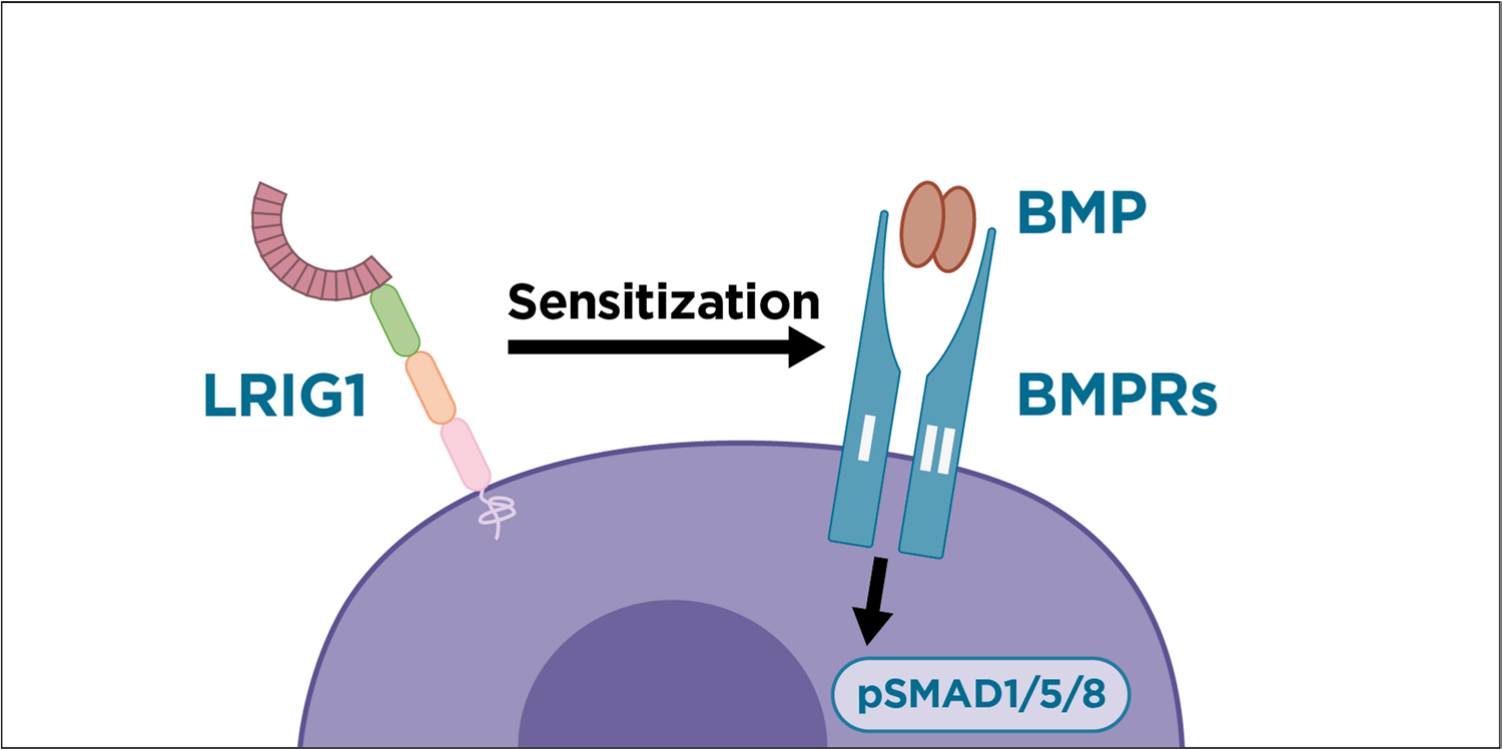
LRIG1 sensitizes cells to BMP signaling. Specifically, LRIG1 (and LRIG3) sensitizes stem cells to BMP signaling through a molecular mechanism that is not yet fully understood. Notably, BMP4 and BMP6 signaling is dependent on LRIG proteins, whereas BMP9 signaling is not [10]. These and other available data have suggested that LRIG1 activity is mediated via BMP type 1 receptor(s) [10, 40]

To date, the function of mammalian LRIG1 has been primarily considered to be the negative regulation of various receptor tyrosine kinases, including members of the EGFR family [36, 37], MET [38], RET [39], and PDGFRA [40]. However, the mechanisms involved and the universality of this function remain unclear. Interestingly, *Lrig*-null MEFs have recently been shown to display normal receptor tyrosine kinase signaling activity [10]. On the other hand, *Lrig*-null MEFs have been shown to be profoundly deficient in BMP signaling capacity [10]. Notably, the expression of LRIG1 or LRIG3 restores BMP signaling that had been inhibited, i.e., both LRIG1 and LRIG3 sensitize cells to signaling induced by BMP4 and BMP6 [10]. However, LRIG2 does not exhibit this BMP-signaling-promoting function. Findings showing that mammalian LRIG1 and LRIG3 promote BMP signaling are in line with previous results obtained with the nematode *Caenorhabditis elegans* showing that the sole *C. elegans* LRIG homolog, SMA-10, regulates worm body size by promoting BMP signaling [41, 42]. The functional relationship between mammalian LRIG1 and LRIG3 and BMP signaling has been further supported by genetic cell proliferation codependency data available through the Dependency Map portal, which comprises whole-genome codependency data of more than 1,000 human cancer cell lines (https://depmap.org). In this dataset, cell lines that show a positive or negative proliferation dependency on LRIG1 or LRIG3 also show significant dependence on genes enriched in BMP signaling pathways (Tables S1 and S3). Notably, no enrichment of genes encoding receptor tyrosine kinases or their signaling pathway components have been identified among LRIG1-dependent or LRIG3-dependent cell lines. LRIG2 does not show a codependent pattern involving either BMP signaling mediators or receptor tyrosine kinase signaling mediators (Table S2), which is consistent with evidence suggesting that LRIG2 exhibits a different molecular function than LRIG1 and LRIG3. The exact molecular mechanism underlying the BMP-signaling-promoting function of LRIG1 and LRIG3 remains to be elucidated. To date, there is no evidence suggesting that LRIG proteins should function by regulating the expression levels of any of the BMP receptors or BMP signaling mediators [10]. The coimmunoprecipitation of ectopically overexpressed proteins suggest that LRIG1 may physically interact with a variety of type 1 and type 2 BMP receptors [39]; however, theses results have not been confirmed in a more physiological setting or by using other methods [28, 43]. Functional results suggest that LRIG protein activity may be mediated through specific BMP-type 1 receptors [1042] (Fig. 1), possibly via the regulation of receptor trafficking [40]. In any case, irrespective of the underlying molecular mechanisms, we hypothesize that the proximal effects of LRIG1 and LRIG3 primarily affect BMP signaling (not receptor tyrosine kinase activity), which has important implications for the regulation of stem cell quiescence.

## Does LRIG1 Regulate Stem Cell Quiescence by Promoting BMP Signaling?

Given the evidence suggesting that LRIG1 is a promoter of BMP signaling, there is a question as to if LRIG1 regulates stem cell quiescence via the regulation of BMP signaling and not via other mechanisms. Indeed, in many stem cell niches where LRIG1 marks quiescent stem cells, BMPs promote the quiescence of corresponding stem cell populations, as discussed below.

Among different epidermal stem cell compartments in mice and humans, in the hair follicle stem cells, BMP signaling has been established as being necessary for quiescence maintenance [44, 45]. However, among interfollicular stem cells in which Lrig1 has been shown to play a regulatory role [18], the role played by BMP signaling has, to our knowledge, not been thoroughly investigated. Nevertheless, analogous to the regulation of hair follicle stem cells and based on the previously demonstrated BMP-sensitizing function of LRIG1, we suggest that Lrig1 plausibly maintains interfollicular skin stem cells in a quiescent state via the promotion of BMP signaling.

BMP signaling plays an important role in intestinal homeostasis. Germline mutations in the human BMP-type 1 receptor gene *BMPR1A* or BMP/transforming growth factor-β signaling-mediator gene *SMAD4* cause juvenile polyposis syndrome and colorectal cancer [46, 47]. In mice, both the conditional inactivation of *Bmpr1a* and the ectopic overexpression of the BMP inhibitor Noggin result in the expansion of the intestinal stem cell compartment and overgrowth of the intestinal epithelium [48–50]. Although Lgr5 + intestinal stem cells cycle and are not quiescent, BMP signaling seems to inhibit their proliferation. Hence, although the existence and nature of putative quiescent intestinal stem cells remain controversial (e.g., [12,18]), BMP signaling seems to inhibit intestinal stem cell proliferation, consistent with LRIG1 suppressing intestinal stem cell proliferation through the regulation of BMP signaling.

In the mouse brain, BMPs are required for slowing the rapid proliferation of embryonal neural precursor cells and inducing the quiescent state of adult neural stem cells [reviewed in, 14]. Additionally, adult neural stem cell activity is regulated by BMPs; for example, BMP2 suppresses both the activation of quiescent neural stem cells and EGF-driven proliferation of transit-amplifying progenitor cells [51]. Interestingly, the suppressive effect of BMP2 is strongly dominant over the stimulatory effect of EGF [51], which is consistent with a model in which BMPs have been shown to be important for the induction and maintenance of the quiescent state and in which EGF induces transit-amplification of progenitor cells in the absence of BMP. Additionally, in zebrafish, BMP signaling is required for adult neural stem cell quiescence mediated via the upregulation of *Id1* expression [52]. Notably, the discovery of Lrig1 as a candidate neural stem cell marker in mice was made by virtue of its expression in an Id1^high^ neural stem cell population *in vitro* [30]. *Id1* is a prototypical BMP-responsive gene [53–55], suggesting that these Lrig1-expressing neural stem cells display an activated BMP signaling pathway. Additionally, *in vitro*, BMPs can induce reversible quiescence of proliferating neural stem cells [56, 57]. In the *in vitro* experimental system employed by Marqués-Torrejón et al., BMP4 drove adult neural stem cells into quiescence [31]. Based on the observations that EGFR inhibitors reverse the excessive proliferation of *Lrig1*-ablated neural stem cells, both Jeong et al. and Marqués-Torrejón et al. concluded that Lrig1 controlled the entry and exit of cells in quiescent and proliferative states through the regulation of EGFR [29, 31]. However, these authors did not consider the recent evidence showing that LRIG1 promotes BMP signaling [10]. With this new knowledge, we alternatively propose that the observed LRIG1-dependent regulation of adult neural stem cell quiescence is mediated by enhanced BMP signaling, which, however, does not exclude an important pro-mitogenic role played by EGFR in these cells.

Less is known about the role of BMP signaling in the regulation of proliferation and quiescence in stem cells of the oral hard palate and mouse incisors. However, given the important role played by Lrig1 in maintaining stem cell quiescence in the oral hard palate and the demonstrated BMP-signaling-promoting function of LRIG1, we propose that quiescence of the stem cells in the oral mucosa is maintained via Lrig1-mediated cell sensitization to BMP signaling. In mouse incisors, BMP signaling plays an important role in the regulation of mesenchymal stem cells [58]; however, the specific role played by BMP signaling in the quiescence of the Lrig1-positive mesenchymal stem cells has not yet been specifically addressed. Similarly, we are unaware of any study investigating the role played by BMP signaling in the quiescence of planarian neoblasts. Nevertheless, based on the discussion above, we speculate that LRIG1 might regulate stem cell quiescence in the oral mucosa, mouse incisors, and planarian neoblasts through the promotion of BMP signaling. However, this hypothesis needs further experimental verification.

## Conclusions

**Fig. 2:**
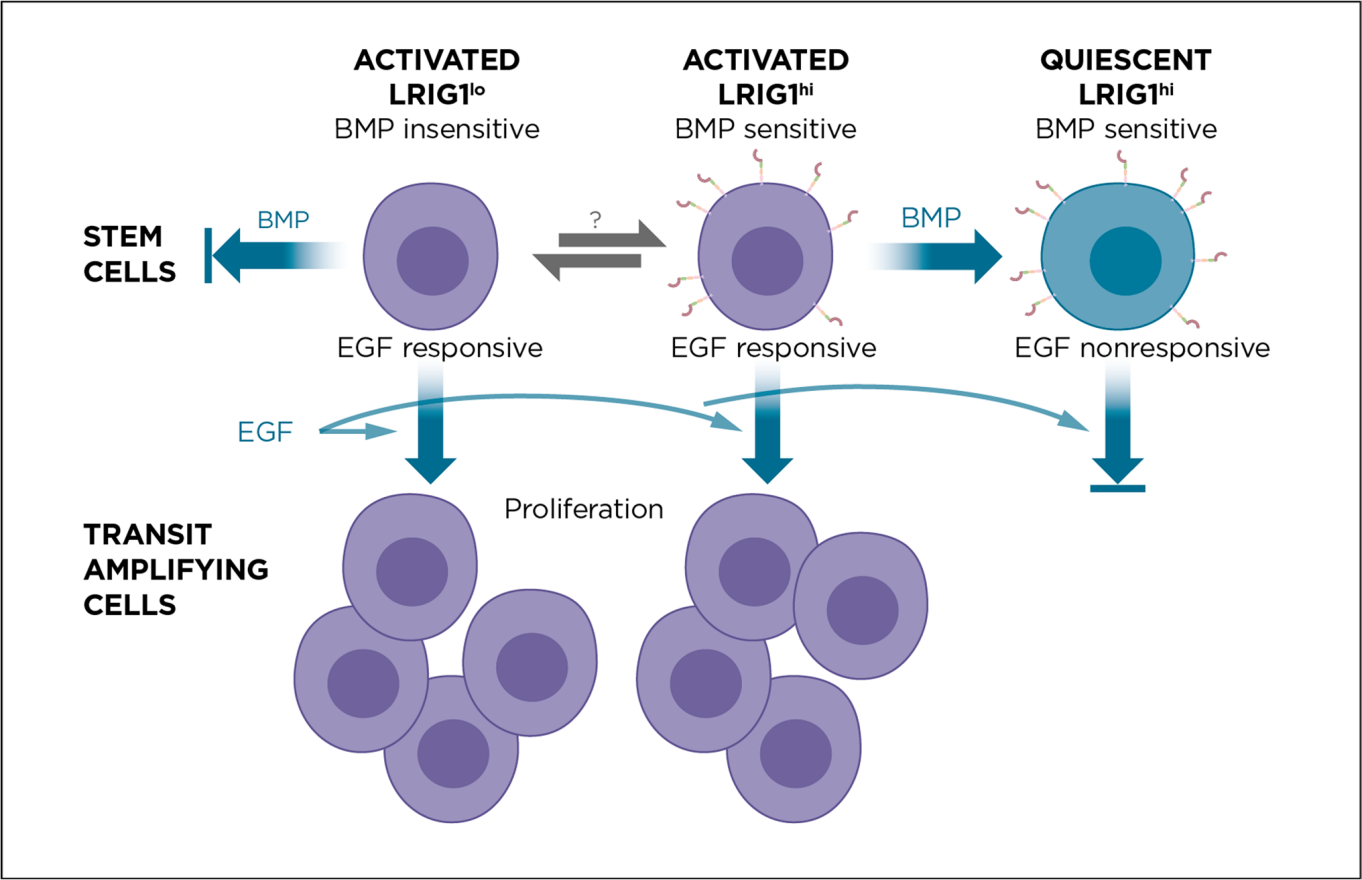
LRIG1 marks and regulates quiescent stem cells via its BMP-sensitizing function. The image shows the hypothesized roles played by LRIG1, BMP, and EGF in adult neural stem cell quiescence and proliferation, which is exemplary of the stem cell niches discussed in the text. Similar schemes can be envisioned for the other stem cell compartments discussed. BMPs induce quiescence in activated LRIG1-positive (LRIG1^hi^) stem cells, whereas LRIG1-negative (LRIG1^lo^) stem cells are largely insensitive to BMP ligands. Quiescent stem cells do not respond to EGF, whereas ‘activated’ stem cells proliferate in response to EGF, thereby forming transit-amplifying progenitor cell populations. Whether and how stem cells transition between the LRIG1^lo^ and LRIG1^hi^ states remain unknown

Considering the recent findings discussed in this review, we hypothesize that LRIG1 regulates stem cell quiescence primarily by promoting BMP signaling, in contrast or in addition to other mechanisms (Fig. 2). Although we do not dispute the importance of EGFR signaling in the regulation of cell proliferation or the possible direct or indirect regulation of EGFR signaling by LRIG1, we propose an alternative model to account for the associations observed between LRIG1 expression and stem cell quiescence. Given the important roles played by BMP signaling in the induction and maintenance of stem cell quiescence and the recent evidence showing that LRIG1 sensitizes cells to BMP signaling, we suggest that the primary effect of LRIG1 on stem cell quiescence is mediated through cell sensitization to BMP signaling, not through other mechanisms. Importantly, this hypothesis is testable. For example, if the hypothesis is correct, the effects of the genetic downregulation of LRIG1 expression on a certain stem cell population will be reversed by a corresponding increase in BMP signaling strength induced by other means; for example, it an be reversed by increasing BMP ligand levels or by reducing BMP inhibitor concentrations, similar to the genetic interplay between Lrig3 and the BMP inhibitor netrin-1 [59] in the formation of the inner ear [60]. Furthermore, because molecular functions are agnostic with regard to cell fates, we can postulate that LRIG1 regulates stem cell quiescence only to the extent that BMP signaling affects quiescence. However, we envision that LRIG1 also promotes other BMP-dependent physiological processes, as has been shown by the role played by LRIG1 and BMP in adipogenesis [10]. In addition, it will be interesting to see whether LRIG3 regulates stem cell quiescence in a manner similar to that of LRIG1, for example, during craniofacial development, which is dependent on proper BMP signaling in suture stem cells [61] and is defective in *Lrig3*-deficient mice [60]. Overall, we suggest that the BMP-signaling-promoting functions of LRIG1 and LRIG3 should be considered when interpreting new and old data regarding the role played by LRIG proteins in stem cell biology and other developmental and physiological processes.

## Supplementary Information

Below is the link to the electronic supplementary material.

Supplementary file1 (DOCX 34.1 KB)

## Data Availability

Not applicable.
