# Supplementary material for: HYPOTHESIS: Do LRIG Proteins Regulate Stem Cell Quiescence by Promoting BMP Signaling?
Source: Stem Cell Rev Rep. 2022 Aug 15;19(1):59–66. doi: 10.1007/s12015-022-10442-9 (PMC9823064; doi:10.1007/s12015-022-10442-9)
Supplement: Supplementary file 1 — Supplementary file1 (DOCX 34.1 KB) [file 12015_2022_10442_MOESM1_ESM.docx]

**SUPPLEMENTARY TABLES**

**Table S1.** The 20 genes showing the highest LRIG1 codependency, as determined through CRISPR technology and retrieved from the Dependency map portal (https://depmap.org/portal/gene/LRIG1?tab=overview). Genes manually assigned as being BMP signaling-associated genes are indicated in bold.

| **Gene** | **Entrez Id** | **Pearson correlation coefficient** |
| --- | --- | --- |
| **MIB1** | 57534 | -0.221 |
| **SMAD7** | 4092 | -0.206 |
| **BMPR1A** | 657 | 0.191 |
| NPM1 | 4869 | -0.181 |
| SLC22A16 | 85413 | -0.181 |
| UBA7 | 7318 | 0.176 |
| LAT | 27040 | -0.173 |
| SP5 | 389058 | 0.172 |
| **SMAD6** | 4091 | -0.170 |
| **RGMB** | 285704 | 0.170 |
| PROK2 | 60675 | 0.163 |
| MBLAC2 | 153364 | 0.163 |
| **SMAD4** | 4089 | 0.163 |
| FGL2 | 10875 | 0.163 |
| SLC6A2 | 6530 | 0.162 |
| RHOBTB1 | 9886 | 0.162 |
| CGGBP1 | 8545 | 0.161 |
| PPM1M | 132160 | 0.160 |
| TMEM158 | 25907 | 0.160 |
| GCG | 2641 | 0.159 |

**Table S2.** The 20 genes showing the highest LRIG2 codependency, as determined through CRISPR technology and retrieved from the Dependency map portal (https://depmap.org/portal/gene/LRIG2?tab=overview). None of the genes were assigned as being BMP signaling-associated genes.

| **Gene** | **Entrez Id** | **Pearson correlation coefficient** |
| --- | --- | --- |
| C1orf162 | 128346 | 0.378 |
| INKA2 | 55924 | 0.377 |
| AMPD1 | 270 | 0.336 |
| SPAG17 | 200162 | 0.321 |
| BCL2L15 | 440603 | 0.304 |
| CYB561D1 | 284613 | 0.302 |
| SORT1 | 6272 | 0.301 |
| PTGFRN | 5738 | 0.293 |
| MAGI3 | 260425 | 0.291 |
| SYPL2 | 284612 | 0.287 |
| GPSM2 | 29899 | 0.284 |
| MOV10 | 4343 | 0.283 |
| WDR63 | 126820 | 0.281 |
| HAO2 | 51179 | 0.280 |
| CD101 | 9398 | 0.279 |
| C1orf52 | 148423 | 0.278 |
| UBL4B | 164153 | 0.276 |
| EVI5 | 7813 | 0.273 |
| TBX15 | 6913 | 0.270 |
| TENT5C | 54855 | 0.269 |

**Table S3.** The 20 genes showing the highest LRIG3 codependency, as determined through CRISPR technology and retrieved from the Dependency map portal (https://depmap.org/portal/gene/LRIG3?tab=overview). Genes manually assigned as being BMP signaling-associated genes are indicated in bold.

| **Gene** | **Entrez Id** | **Pearson correlation coefficient** |
| --- | --- | --- |
| **RGMB** | 285704 | 0.263 |
| **SMAD7** | 4092 | -0.233 |
| **BMPR1A** | 657 | 0.221 |
| **SMAD4** | 4089 | 0.216 |
| R3HDM2 | 22864 | 0.210 |
| OS9 | 10956 | 0.209 |
| PHLDA1 | 22822 | 0.208 |
| SLC16A7 | 9194 | 0.201 |
| **BAMBI** | 25805 | -0.199 |
| GRIP1 | 23426 | 0.198 |
| HOXC11 | 3227 | 0.197 |
| **IPO8** | 10526 | 0.195 |
| MAP3K12 | 7786 | 0.194 |
| LACRT | 90070 | 0.192 |
| FAM186A | 121006 | 0.192 |
| STAC3 | 246329 | 0.187 |
| RAPGEF2 | 9693 | 0.184 |
| ESYT1 | 23344 | 0.181 |
| **MIB1** | 57534 | -0.181 |
